# Social determinants of eyeblinks in adult male macaques

**DOI:** 10.1038/srep38686

**Published:** 2016-12-06

**Authors:** Sébastien Ballesta, Clayton P. Mosher, Jeno Szep, Kate D. Fischl, Katalin M. Gothard

**Affiliations:** 1 Department of Physiology, College of Medicine, The University of Arizona, Tucson, AZ, 85724, USA; 2 Department of Electrical and Computer Engineering, Johns Hopkins University, Baltimore, MD, USA

## Abstract

Videos with rich social and emotional content elicit natural social behaviors in primates. Indeed, while watching videos of conspecifics, monkeys engage in eye contact, gaze follow, and reciprocate facial expressions. We hypothesized that the frequency and timing of eyeblinks also depends on the social signals contained in videos. We monitored the eyeblinks of four male adult macaques while they watched videos of conspecifics displaying facial expressions with direct or averted gaze. The instantaneous blink rate of all four animals decreased during videos. The temporal synchrony of blinking, however, increased in response to segments depicting appeasing or aggressive facial expressions directed at the viewer. Two of the four monkeys, who systematically reciprocated the direct gaze of the stimulus monkeys, also showed eyeblink entrainment, a temporal coordination of blinking between social partners engaged in dyadic interactions. Together, our results suggest that in macaques, as in humans, blinking depends not only on the physiological imperative to protect the eyes and spread a film of tears over the cornea, but also on several socio-emotional factors.

Blinking serves multiple purposes. The reflexive closure of the eyelids maintains the moisture of the cornea and protects the eyes from foreign objects^1^,^2^,^3^,^4^. The rate and timing of the eyeblinks, however, does not merely reflect the physiological status of the eyes. In both humans and non-human primates, blinking has been linked to cognitive states and to social engagement with conspecifics^5^,^6^,^7^,^8^,^9^,^10^,^11^.

Eyeblinks also play a role in social communication. Indeed, humans often attribute mental states to their social partners based on observed changes in their blinking behavior^12^,^13^,^14^. Furthermore, humans coordinate the timing of their blinks with the blinks of their social partners^15^,^16^. This phenomenon, called *eyeblink entrainment*, is absent when the social partners are prevented from fully engaging with each other (e.g., seeing each other speak without any audio to convey the message)^15^,^16^,^17^. Such observations suggest that eyeblink entrainment is not an automatic imitation of blinking but an elemental form of social interaction.

Macaque monkeys may also entrain their eyeblinks to one another during real-life dyadic social interactions. It is unknown whether videos of natural social behaviors, constructed to serve as a proxy for dyadic social interactions, can induce eyeblink entrainment in viewer monkeys. Previous studies have shown that videos with social content induce several interactive social behaviors, such as gaze following, the reciprocation of eye contact and facial expressions^18^,^19^,^20^,^21^. Videos depicting social stimuli, however, cannot fully substitute real-life interactions because they are limited by a major shortcoming: the behavior of the stimulus monkey remains unchanged despite the viewer’s attempt to respond to the perceived social signals and engage the protagonist. Nevertheless, videos are valuable stimuli for neurophysiological studies because they can be presented multiple times and their presentation can be coupled with both non-invasive physiological monitoring (e.g., eye tracking, autonomic recordings) and invasive measures of brain activity (intracranial recordings). If the ultimate goal is to understand the neural events that govern social behavior in primates, it is critical to use the most adequate stimuli to elicit mental states in laboratory settings that closely resemble the mental states in real-life dyadic interactions.

The aim of the current study was to determine the social factors that predict when monkeys blink while they view videos of natural social behaviors displayed by unfamiliar conspecifics. Based on previous observations that monkeys display natural social behaviors toward monkeys shown in videos^18^,^19^,^20^,^21^,^22^, as though they are attempting to socially engage them, we hypothesized that the blinking behavior in response to videos would be comparable to blinking behavior during real-life social interactions. We predicted that monkeys would entrain their eyeblinks while watching videos, just as humans entrain their eye blinks during real life social interactions. We further expected their blink frequency to be modulated by the emotional expressions of their social partners.

## Results

Four male monkeys, QT, RI, RU, and ZI viewed 178, 130, 143 and 330 unique 10s long videos respectively over a total of 62 recording sessions (QT = 16 sessions, RI = 10 sessions, RU = 13 sessions, ZI = 23 sessions). The majority of the videos depicted unfamiliar monkeys (henceforth stimulus monkeys), placed in a plexiglass cage where they displayed socially meaningful facial expressions, postures, and gestures. Most of these videos depicted only one monkey, but a subset of these videos showed 2 or more monkeys (13% of video exposures). We also displayed videos of individual monkeys or groups of monkeys in natural outdoor settings. These video segments were recorded in the field station of the California National Primate Research Center and on the field station of Cayo Santiago. The segments were not explicitly chosen to show facial expressions, but on occasion facial expressions are visible. Of the 367 videos, 99 videos clips were seen by all four monkeys. Each monkey viewed each video 3–15 times.

**Figure 1:**
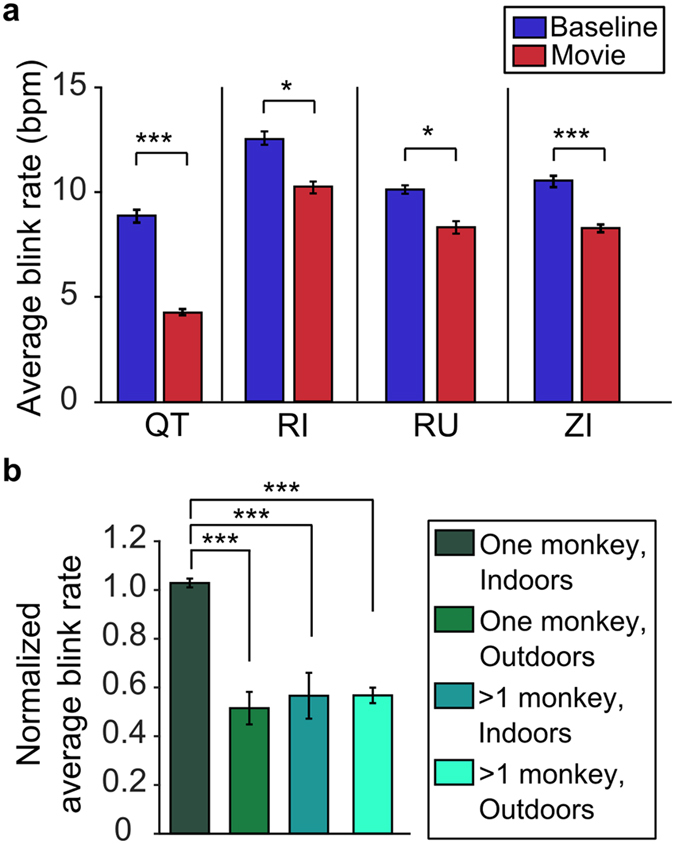
Video watching lowered the blinking rate of the viewer monkeys. (**a**) Average + SEM blink rate (blinks per minute = bpm) during video viewing (red bars) compared to baseline (blue bars). Each of the four monkeys blinked significantly less when viewing movies (Wilcoxon rank-sum test, QT: p = 1.86 × 10^−6^, RI: p = 0.011, RU: p = 0.040, ZI: p = 1.84 × 10^−5^). (**b**) Eyeblink rate depends on the social complexity of the movie content. Eyeblink rates have been normalized to the average blink rate during video viewing of each monkey. The blinking rates during videos of different content were compared using a Wilcoxon two-tailed rank-sum test. Asterisks indicate significant differences. Eyeblink rate decreased significantly during videos that occurred in natural settings and videos that depicted multiple monkeys, p = 4.37 × 10^−8^, p = 1.76 × 10^−4^, p = 3.10 × 10^−19^, respectively as graphically displayed. For both (**a**,**b**) *p < 0.05, ***p < 0.001, Wilcoxon rank-sum test. Error bars represent SEM.

Eyeblink rate decreased significantly when the monkeys watched the videos (Wilcoxon rank-sum test, on averages/session, *QT: p* = *1*.*86* × *10*^−*6*^, *RI: p* = *0*.*011*, *RU: p* = *0*.*040*, *ZI: p* = *1. 84* × *10*^−*5*^; Fig. 1a, compared to baseline period when monkeys viewed a blank screen). The reduction in blink rate correlated with the content of the videos (Fig. 1b). Videos depicting more than one monkey or monkeys in outdoor environments induced a larger decrease in eyeblink rate than videos depicting a single monkey in an indoor environment (Wilcoxon rank-sum test, p = *4*.*37* × *10*^−*8*^, (one monkey indoors vs. one monkey outdoors); *p* = *1*.*76* × *10*^−*4*^ (one monkey indoors vs. multiple monkeys indoors), and *p=3*.*10* × *10*^−*19*^ (one monkey indoors vs. multiple monkeys outdoors) (Fig. 1b). We observed no significant differences among movies that depicted more than one monkey indoors, one monkey outdoors, or multiple monkeys outdoors (Wilcoxon rank-sum test, one monkey outdoors vs. multiple monkeys indoors: p = 0.70, one monkey outdoors vs. multiple monkeys outdoors: p = 0.30 and multiple monkey indoors vs. multiple monkeys outdoors p = 0.71) (Fig. 1b).

**Figure 2:**
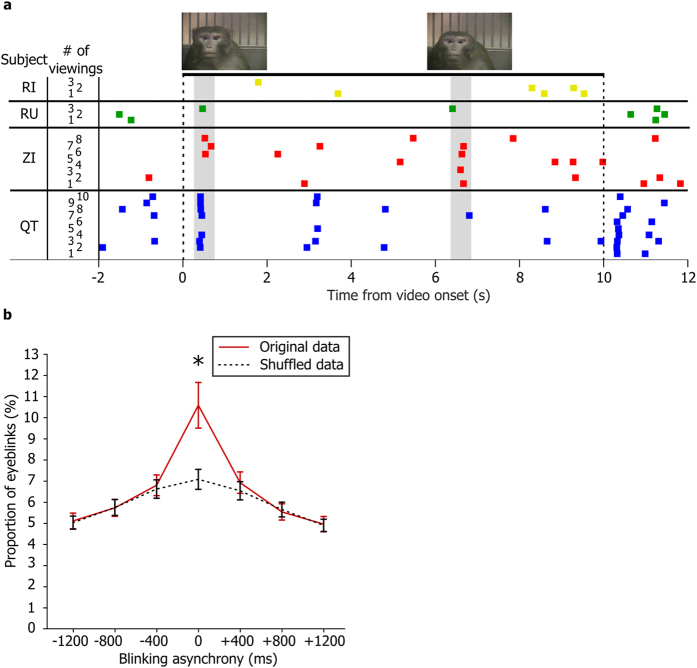
Eyeblink clustering across repeated viewings of the same video. (**a**) The colored squares represent the eyeblinks of the 4 viewer monkeys on each trial (blue = monkey QT, red = monkey ZI, green = monkey RU, and yellow = monkey RI). The dotted vertical lines represent the beginning and the end of the video. Frames from this video show the behavior of the stimulus monkey immediately prior to the cluster of blinks (marked by gray bars). The blinking probability of 3 of the 4 viewers increased in response to two time segments in this video. In the first segment the stimulus monkey stared insistently at the viewer, a behavior considered as an assertion of dominance or covert threat. The second cluster of blinks occurred when the same animal began displaying a lipsmacking (appeasing) expression with direct gaze. At this time in the video the stimulus monkey also blinked. Note that monkey QT systematically blinked after the presentation of the videos (clustering of blue marks at the termination of the video). (**b**) Probability of eyeblink clustering across all four subjects, based on the viewing of 1,615 videos. The solid red line represents the proportion of eyeblinks that occurred within windows of a time of 400 ms during repeated presentations of the same video. The dashed line represents the same proportion for shuffled eyeblink data (see methods). The difference between the two curves reflects the degree of eyeblink synchronization (*two-way ANOVA on 7 bins: p = 0.024 (F = 5.06) for shuffling, p = 9.37 × 10^−18^ (F = 15.5) for asynchrony and p = 0.0015 (F = 3.60) for interaction). The post hoc two-tailed t-test at the central bin showed a significant difference (p = 0.0030) between the actual and shuffled data. No significant difference was found at any of the other time bins. Error bars represent SEM.

We next explored how the occurrence of viewers’ eyeblinks correlated with the unfolding of the stimulus monkeys’ socio-emotional behaviors. We found that, even though the viewer monkey’s blink rate was reduced during video viewing compared to baseline, the eye blinks appeared with higher probability at particular moments during the viewings. The blinking of the viewers clustered across multiple viewings of the same video (Fig. 2a), suggesting that blink rate was related to the visual and/or socio-emotional content of the videos. This clustering appeared both for repeated viewings by the same monkey and across monkeys. Indeed, the probability of blinking in a window of 400 ms (±200 ms from the blink in a different viewing) was higher than chance (Two-way ANOVA on 7 bins: *p* = *0*.*024 (F* = *5*.*06*) for shuffling, *p* = *9*.*37* × *10*^−*18*^ (*F* = *15*.*5*) for asynchrony and *p* = *0*.*0015 (F* = *3*.*60*) for interaction. The post hoc two-tailed t-test at the central bin showed a significant difference (*p* = *0*.*0030*) between the actual and shuffled data. No significant difference was found at any of the other time bins; Fig. 2b).

This synchronization of blinking across trials and among monkeys is unlikely to be due to low-level visual features. Indeed, the probability of blinking was not significantly correlated with the amount of motion in the videos (quantified at pixel-by-pixel changes in brightness) (Spearman rank correlation, QT, R = −0.035 (*p* = *0*.*44*), ZI, R = −0.061 (*p* = *0*.*18*), RU, R = −0.050 (*p* = *0*.*27*), RI, R = 0.021 (*p* = *0*.*64*). Rather, the increases in blink synchrony appear to be the result of the socio-emotional content of the videos.

**Figure 3:**
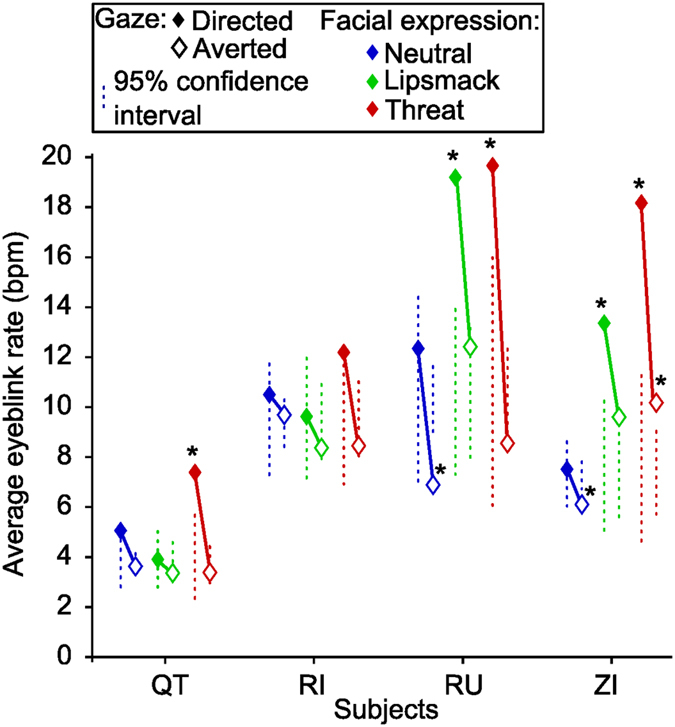
Viewer monkeys blink more frequently in response to facial expressions with direct gaze. The average eyeblink rate of each viewer monkey was calculated during epochs when the stimulus monkey displayed a facial expression (neutral, lipsmack, or threat) and directed or averted its gaze toward or away from the viewer. Each vertical dotted line represents the 95% confidence interval calculated from shuffled data (see methods). The diamonds indicate the mean value of the eyeblink rate. Neutral faces (in blue) with either directed (filled diamonds) or averted gaze (open diamonds) did not elevate the blinking rate above the value expected by chance. Threatening (antagonistic) and lip-smacking (affiliative) expressions however, significantly elevated the blinking rate of the viewer (permutation test, p < 0.05) with the exception of monkey RI who did not respond to any facial expressions with additional blinking. Asterisks refer to values that are outside the 95% confidence interval.

To identify the specific behavioral events that might cause the clustering of eyeblinks, we explored the relationship between the social signals emitted by the stimulus monkey (gaze direction, facial expression) and blinking behavior of the viewer monkey. We found that the viewer monkeys blinked more frequently when the stimulus monkey displayed a facial expression directed at the viewer monkey (permutation test, p < 0.05, for the specific of this test, see methods; Fig. 3). All four monkeys showed a tendency to blink more frequently when the stimulus monkey’s gaze was directed at the viewer. However, this increase in blink rate depended on the facial expression of the stimulus monkey. Three of the 4 monkeys blinked more often while looking at the direct gaze of a stimulus monkey with a threatening facial expression; two of the 4 monkeys blinked more often while looking at the direct gaze of a stimulus monkey with an appeasing facial expression (permutation test, p < 0.05, see methods; Fig. 3).

**Figure 4:**
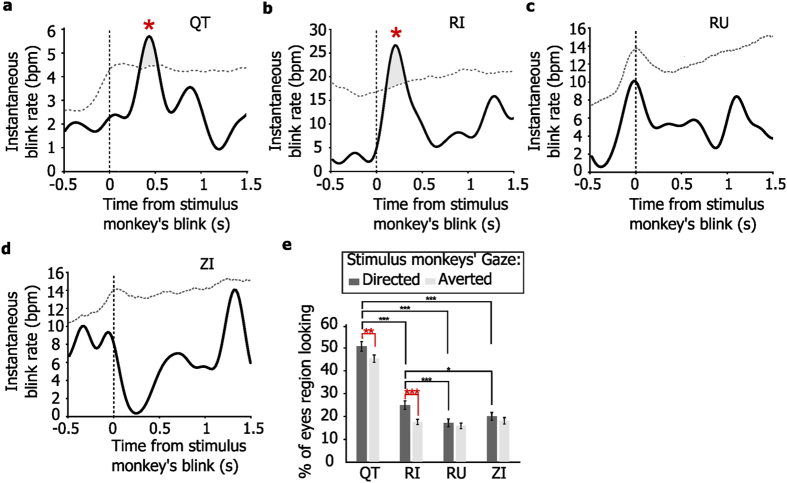
Eyeblink entrainment induced by videos with social content. Each line plot (**a**–**d**) shows the average instantaneous blink rate of the four viewer monkeys aligned to the eyeblinks of the stimulus monkey. The vertical dotted line (time zero) represents the eyeblinks of the stimulus monkey. The horizontal dotted curve represents the boundary of the 95% confidence interval for blink rate calculated from shuffled data (see methods). Asterisk indicates significant (p < 0.05) increases in blinking rate. (**d**) Monkeys QT and RI looked longer at the eye regions of the stimulus monkeys with directed gaze (eye contact) than with averted gaze. RU and ZI, however did not look longer at eyes with direct gaze and looked less at the eyes overall compared to QT and RI. (*p < 0.05, **p < 0.01, ***p < 0.001, Wilcoxon signed rank test). Error bars represent SEM. Blink entrainment occurred within 500 ms after the eyeblinks of the stimulus monkey.

Finally we calculated the temporal relationship between the blinking of the stimulus monkey and the blinking of the viewer monkeys. This phenomenon, called eyeblink entrainment, requires the viewer to blink concurrently with its social partner (within 500 ms). Two of the four monkeys (QT and RI) entrained their eyeblinks to the stimulus monkey’s blinks (permutation test, p < 0.05; Fig. 4a and b). The other two subjects (RU and ZI) did not entrain their eyeblinks (the blinking rate of these monkeys did not exceed levels expected by chance, where chance values are based on a 95% confidence interval based on shuffled data; Fig. 4c and d). Monkeys RU and ZI were also less likely to look at the eyes of the stimulus monkey (Wilcoxon signed-rank test: QT vs. RI *p* = *1*.*68* × *10*^−*19*^; QT vs. RU *p* = *9*.*05* × *10*^−*23*^; QT vs. ZI *p* = *2*.*08* × *10*^−*21*^; RI vs. RU *p* = *2*.*09* × *10*^−*4*^; RI vs. ZI *p* = *0*.*029*; RU vs. ZI *p* = *0*.*052*; Fig. 4e). In contrast, monkeys QT and RI, who showed eyeblink entrainment, reciprocated eye contact, by looking longer at the directed rather than at the averted eyes of the stimulus monkeys (Wilcoxon signed-rank test: QT *p* = *0*.*001*, RI *p* = *3*.*98* × *10*^−*5*^, RU *p* = *0*.*76*, and ZI *p* = *0*.*29*; Fig. 4e).

## Discussion

We examined the blinking behavior of four monkeys while they viewed videos of conspecifics displaying facial expressions with directed or averted gaze. We found that all four monkeys blinked less during the presentation of videos than during baseline periods. Even though monkeys blinked less during videos, their blinks became more temporally aligned to specific events in the video such as the production of facial expressions and the blinking of the stimulus monkeys.

During eyeblinks visual input is interrupted for about 200 ms^23^. A voluntary suppression of blinking might thus indicate a need to increase the gathering of visual information^9^,^22^. Indeed, we observed a reduction of blinking during the videos relative to the baseline. This reduction in blinking was strongest when monkeys viewed videos of multiple monkeys in natural social settings. It is likely that the more visually rich videos better captured the viewer’s attention. This interpretation is congruent with findings that show an inverse relationship between blinking rate and attention in humans^9^,^24^.

The observed increases in eyeblink rates in response to facial expressions might reflect a process of overriding attentional needs by ongoing socio-emotional processes. Judicious social decisions require monkeys to process quickly and efficiently large amounts of visual information. Closing the eyes, even for the duration of an eyeblink, has been shown to help cope with increased cognitive load^25^,^26^. This might explain the significant increase in blinking rate that occurred in response to the segments of the video in which the stimulus monkeys displayed threatening or appeasing facial expressions directed at the viewer. It is also possible that blinking in these situations reduces not only processing demands, but the subjective, emotional impact of these potent social signals. The observation that different viewer monkeys tend to blink in response to the same video segment supports the idea that blinks might punctuate the flow of information during socially meaningful interactions^27^.

Three of the four monkeys increased their eyeblink rate in response to threatening or appeasing facial expressions with direct gaze. The blink rate of the fourth monkeys was just marginally significant (at 96.4%, where 97.5% is the upper limit of the two-tailed test). Averted gaze, did not cause a similar increase in blink rate in any of the four monkeys, suggesting that direct gaze has a stronger effect on social behavior than averted gaze^11^ enabling either social avoidance or approach^28^. This is also consistent with the finding that direct gaze activates, in the amygdala, a set of neurons singularly tuned to eye contact^20^ and that patients with amygdala damage rarely make eye contact during face-to-face social interactions^29^. The biological basis and the potential functions of these changes in blinking behavior during social contact remain to be elucidated.

The eyeblink entrainment reported here is highly similar to the eyeblink entrainment reported in humans^15^,^16^. In humans, eyeblink entrainment is not a mere imitation of the blinks of others^15^ rather, it is considered a marker of ongoing, fully-engaged social interactions. It follows, therefore, that at least two of the subject monkeys were socially engaged with the perceived social partner in the videos. Indeed, the two monkeys that showed eyeblink entrainment also looked longer at the eyes of the stimulus monkeys, reciprocating more often their direct gaze. Looking insistently at the eyes and returning eye contact are indicative of dominant social status in macaque societies^30^. The failure of the viewer monkeys to reciprocate the blinks of their social partner might therefore represent an active form of avoiding social engagement with a dominant individual. Individual variations in eyeblink entrainment may thus be considered as a measure of the viewer’s subjective assessment of his or her status relative to the social partner. It would be interesting to determine whether the timing and rate of eyeblinks during social interactions could be added to the list of behaviors currently used for status and personality assessments in monkeys^31^,^32^,^33^,^34^.

In summary, macaques not unlike humans, blink less while they visually attend to eventful videos^6^,^9^,^24^. While the global rate of blinking was reduced, the timing of the blinks appeared to mark events in the video that carried significant social weight^10^,^27^. Interestingly, monkeys also showed blink entrainment, as an elemental form of social engagement^15^,^16^. These findings support the view that blinking behavior of monkeys, particularly during social interactions, can be used as a measure of the ongoing socio-cognitive states.

## Methods

### Subjects and stimuli

Behavioral data were collected from four adult male rhesus macaques (*Macaca mulatta*): QT, RI, RU, and ZI. At the time of the study the ages of all animals varied between 6 and 12 years. Monkeys were housed in double-size cages in the same room with visual access to all other monkeys in the colony. All experiments were performed in compliance with the guidelines of the National Institute of Health for the use of primates in research and were approved by the Institutional Animal Care and Use Committee at the University of Arizona. For accurate eye tracking each monkey was fitted with a head-fixation ring, which attached at three points to titanium pins embedded in an implant. The implant was attached to the skull by a surgical procedure under isoflurane anesthesia. Subject monkeys were seated in custom built primate chairs with their eyes positioned 57 cm from an LCD monitor spanning 37 × 28 degrees of visual angle (dva). Videos subtended 26 × 18 dva, contained 299 frames shown at 30 frames per second and contained no cuts. Neurobehavioral Systems Presentation software (Albany, CA) was used for the display of the videos. Prior to each experimental session monkeys were calibrated by fixating on a nine-point calibration grid. Errors were within ±1 dva.

The data were collected across 5 years of similar experimental protocols all involving passive viewing of social videos. The duration of each video was 10 s; during this time an unfamiliar monkey (stimulus monkey) displayed at least one or more threatening, neutral, or appeasing facial expressions accompanied by the corresponding postural changes. Each video contained multiple repeats of the same facial expressions with gaze either directed at or averted from the viewer. A trial (the presentation of a video) was preceded by the display of a central visual cue that remained on the monitor for 1,150 ± 250 ms. The presentation of the cue was followed by a 600 ± 200 ms period when the monitor was blank. The animals were not required to maintain their gaze within the boundary of the video to be rewarded. The inter-trial interval was 9.7 ± 3.3 seconds. Under our experimental conditions, it was crucial to exclude from the baseline measurement any task-related burst of eyeblink (e.g. after the presentation of the visual cue that preceded the videos or after the end of the video viewing). We thus calculated the baseline during the long inter-trials intervals, (between 7.5 seconds post-video viewing to 2.5 seconds before the next video viewing) and the intervals between the presentations of blocks of videos that spanned several minutes when the monitor was blank. The video content was ethogrammed frame-by-frame to record direction of gaze (averted or directed at the viewer), eyeblinks, and facial expressions (neutral, lip-smacking or threatening^35^). The ethogram also recorded the number of monkeys in the frame and the background (indoors or outdoors). The videos were recorded in different environments marked in Fig. 1 as “indoors” and “outdoors” for semi-free ranging animals and wild macaques. The frames in which the stimulus monkey’s eyes were more than half-covered by the eyelids were scored as the part of an eyeblink. Fixations on the eye region were classified based on regions-of-interest boundaries manually outlined using custom-written scripts in Matlab R2016A (Mathworks).

### Eyeblink and eye position measurement

Eye position and pupil diameter were recorded using an infrared camera with a sampling frequency of 240 Hz (ISCAN Inc., Woburn, MA) and collected as an analog signal using a CED Power 1401 data acquisition system and Spike 2 software (Cambridge Electronic Devices, UK). Eyeblinks were detected by a custom written script that analyzed pupil diameter. Short, reversible losses of pupil data were identified as eyeblinks (when the eyelids were closed and the pupil was no longer exposed to the infrared beam, and the eye tracking system defaulted to maximum voltage). The pupil diameter data were smoothed with a 15 ms sliding window and a second derivate of the pupil diameter signal was taken to find the deflections (valleys) that corresponded to potential eyeblinks (Fig. S1). The baseline level of the signal prior to each valley was determined to be the lower of the two highest points from either side of the valley within a 200–400 ms window. The depth of the valley was defined as the difference between the baseline and the minimum value of the valley. Two straight lines were fitted to the signal between the one third and the two third point depths on each side of the valley. The duration of the blink was defined as the length of the section between the intersections of the fitted lines with the baseline. Valleys in the signal were considered to be eyeblinks if their duration was in the range of 20–800 ms. The minimum duration between the beginnings of two consecutive eyeblinks was 200 ms. This method was manually verified using a video recording of the viewer monkey’s face, with 94% match between the automated system and manual identification on a random video sample.

### Data analysis

All data analysis and statistics were performed using custom-written scripts in Matlab R2016A (Mathworks). To account for individual differences in blink rates, we calculated the mean blink rate during the movie and during baseline periods. Baseline periods began 7.5 seconds after the termination of each video and ended 2.5 seconds prior to the presentation of the next video. The intervals between blocks of videos, when the monitor was blank, and that typically spanned several minutes, were also included the calculation of baseline blinking rates.

To assess the temporal clustering of blinks among viewers (Fig. 2b), we adopted a method previously used by Nakano and colleagues^27^. Briefly, we calculated the shortest time interval between a blink in a given presentation (reference) and all the other blinks in each different presentation of the same video (test). These time differences were binned into 400 ms bins. The same procedure was then applied to surrogate data obtained by shuffling blink times. The shuffled blink data was obtained by shifting all the blinks within a trial by a random time (with circular boundary conditions). This form of shuffling preserves the natural blink rate of the monkey but disrupts the relationship between the blinks and the content of the videos.

To establish a correlation between the viewer’s blink rate and the stimulus monkey’s facial expression, we marked the frames that contained neutral, appeasing (lip-smacking) and threatening (open-mouth threat) facial expressions. We also marked for each frame the gaze direction of the monkey shown in the video and whether the viewer monkey was looking at the video. We then compared the average blink rate during each expression and gaze direction combination to the blink rate during re-sampled, time-matched video segments. We only included in the analysis video segments when the viewer monkey was gazing at the video. We calculated 2000 shuffled time-matched segments and determined whether the blink rate during each facial expression fell outside the 95% confidence interval (two-tailed test).

Eyeblink entrainment was quantified in two steps. First we calculated the average instantaneous blink rate of the viewer monkey relative to the blinks of the stimulus monkey. The instantaneous blink rate, was calculated based on a formula used previously by Shultz *et al*.^24^:


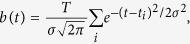


where *b*(*t*) is the time-dependent instantaneous blink rate function and *t*_*i*_ are the blink times. The standard deviation of the Gaussian kernel was chosen as a fixed value of σ = 100 ms^36^. We used T = 60 s to express the results in blink-per-minute (bpm) units.

Second, we determined whether the observed eyeblink entrainment was significantly different than expected by chance, we generated a reference dataset by replacing the blinks of the stimulus monkey with the same number of uniformly distributed randomly generated blinks. This randomization process was repeated 3000 times to yield 3000 different peri-event time histograms. The observed eyeblink entrainment was then compared to the 95% confidence interval calculated from these 3,000 surrogate peri-event time histograms (one-tailed comparison, looking for eyeblink entrainment that was significantly higher than chance).

We included in the analysis only the trials in which the viewer looked at the video for at least 200 ms (the duration of 1–2 fixations) before the stimulus monkey blinked. We included this criterion to be certain that the viewers were attending to the stimulus monkey and thus, noticed the stimulus monkey’s blink. During the first 300 ms in the plot the viewer may or may not be looking at the eyes of the stimulus monkeys. In monkeys that looked frequently at the eyes (e.g., QT), the confidence interval calculated from the shuffled data appears to be low 500 ms before the stimulus monkey’s eye blink and then gradually rises to a stable value by time point 0 ms (Fig. 4a). This is due to our 200 ms video-looking limit (this also explains why the shuffled data/upper limit of the confidence interval is not straight).

Given that monkeys have high levels of blink suppression during the first video viewing, we excluded this trial when analyzing eyeblink entrainment. Likewise, given that monkeys spend less time looking at the videos after several repeated exposures, they are less likely to see the eyeblink of the stimulus monkey. To account for this, we only included trials up to the fifth viewing. We also eliminated from the analysis 10 percent of trials where the viewer monkey spent the most time looking at the screen and 10 percent of trials where the viewer monkey spent the least time looking at the screen (often the last trial).

## Additional Information

**How to cite this article**: Ballesta, S. *et al*. Social determinants of eyeblinks in adult male macaques. *Sci. Rep.* **6**, 38686; doi: 10.1038/srep38686 (2016).

**Publisher's note:** Springer Nature remains neutral with regard to jurisdictional claims in published maps and institutional affiliations.

## Supplementary Material

Supplementary Information
