# Supplementary material for: Social determinants of eyeblinks in adult male macaques
Source: Sci Rep. 2016 Dec 6;6:38686. doi: 10.1038/srep38686 (PMC5138631; doi:10.1038/srep38686)
Supplement: Supplementary Information [file srep38686-s1.pdf]

Supplementary information

Social determinants of spontaneous eyeblinks in adult male macaques

Sebastien Ballesta[1], Clayton P. Mosher[1], Jeno Szep[1], Kate D. Fischl[2], Katalin M. Gothard[1]*

[1] Department of Physiology, College of Medicine, The University of Arizona, Tucson, AZ 85724, USA

[2] Department of Electrical and Computer Engineering, Johns Hopkins University, Baltimore, MD, USA

*corresponding author: gothard94@gmail.com

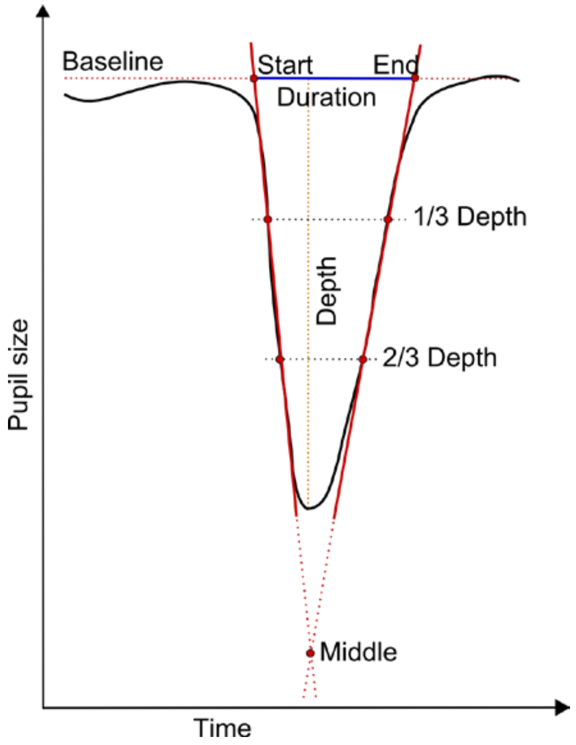

Figure S1. Scheme of parameters used for eyeblink detection*. The baseline level of each eyeblink was determined as the lower of the two highest points from either sides of the valley within a 200-400ms window depending on the subject. The depth of the valley was defined as the difference between the baseline and the minimum value of the valley. Straight lines were fitted to the 1/3 and 2/3 depth of the valley and two fitted lines were drawn through those points. The duration of the eyeblink was defined as the length of the section determined by the intersections of the fitted lines with the baseline. The time of the blink was defined as the time of the deepest point in the valley.*

.
